# Associations Between Sleep, Nutrition, and Health

**DOI:** 10.3390/nu17081360

**Published:** 2025-04-16

**Authors:** Piril Hepsomali, John A. Groeger

**Affiliations:** 1 School of Psychology and Clinical Language Sciences, University of Reading, Reading RG6 6ET, UK; 2 Department of Psychology, School of Social Sciences, Nottingham Trent University, Nottingham NG1 4BU, UK; john.groeger@ntu.ac.uk

## 1. Introduction

Poor sleep is highly prevalent, affecting a significant proportion of the global population, and imposing a substantial burden on individuals’ health, quality of life, and productivity [1,2,3,4]. Its prevalence is shaped by various personal factors, including age, sex, health status, income, and ethnicity, as well as broader societal influences such as social deprivation [5].

Although numerous sleep disorders exist, each with distinct aetiologies, the majority of individuals experience transient episodes of inadequate or poor-quality sleep rather than chronic disruptions. Despite the availability of standard treatments such as pharmacotherapy and cognitive behavioural therapy for insomnia, many individuals continue to experience inadequate relief [6,7]. Furthermore, people typically rely on behavioural change or non-pharmacological treatments for transient sleep challenges. As a result, whether for transient or enduring sleep challenges, people are likely to consider alternative and complementary solutions incorporating lifestyle interventions.

Of specific importance, diet is known to be a modifiable risk factor for metabolic and mental disorders [8,9,10,11] and could also influence sleep [12] through several mechanisms, including its effects on inflammation, the gut–brain axis, and circadian rhythms [13,14,15]. Cross-sectional studies have identified specific nutrients, food items, and dietary patterns that are associated with sleep outcomes [16,17,18,19,20]. Furthermore, interventional research has shown promising results, with targeted dietary changes improving sleep quality and duration in both clinical and general populations [21,22,23].

## 2. An Overview of Published Articles

Advancing research on the relationship between diet and health, as well as exploring dietary approaches to improve well-being, remains a critical scientific priority. The Special Issue (SI) “*Associations between Sleep, Nutrition, and Health*” highlights both established and emerging themes in this field. Through six articles, this SI examines the complex interplay between sleep duration and quality, nutrition, and aspects of health.

The current SI includes a number of reviews exploring the timing of eating (Contributor 1) and the inflammatory potential of diets (Contributor 2). Contributor 1 summarises evidence from human studies on the associations between chrononutrition and cancer risk, with a specific emphasis on potential mechanisms of action, highlighting that early time-restricted eating and prolonged nighttime fasting could be associated with a lower risk of cancer through cell cycle regulation, the modulation of metabolic pathways and inflammation, and gut microbiota fluctuations. Focusing on human cross-sectional studies, Contributor 2 examines the associations between the inflammatory potential of diets through utilising a dietary inflammatory index, sleep duration, and sleep quality, showing that pro-inflammatory diets may be associated with poor sleep duration and quality.

This SI also features an original research article investigating the associations between specific food groups and sleep duration. Conducted in an ethnically diverse, lower-income cohort of midlife and older adults, Contributor 3 found that longer sleep duration was linked to higher fruit and vegetable intake in males, but not in females, highlighting a potential sex difference in the sleep–diet relationship among aging populations.

One recurring theme in this SI concerns the benefits of various dietary compounds on sleep quality and mental well-being (Contributors 4, 5, and 6). Contributor 4 found that while a four-week daily intake of matcha did not objectively improve sleep (through EEG measures of total sleep time, sleep latency, wake after sleep onset, and sleep efficiency), it showed trends toward enhanced self-reported sleep satisfaction and reduced depressive symptoms in healthy adults. Similarly, Contributor 5 demonstrated mood benefits and increased subjective sleep duration with a four-week daily intake of *Bifidobacterium adolescentis* SBT2786 but no effect on subjective sleep quality in healthy adults. Conversely, Contributor 6 reported that a three-month *Aloysia citrodora* (lemon verbena) supplementation significantly improved both subjective and objective sleep quality in poor sleepers, along with increased nocturnal melatonin levels.

## 3. Future Research

The articles in this SI collectively emphasise the crucial relationship between nutrition, sleep, and overall health. They highlight the importance of considering meal timing, diet quality, and sex-specific differences in the sleep–diet connection. Additionally, they underline the potential of various dietary interventions as both alternative and complementary strategies to improve sleep health, offering valuable insights into how tailored nutrition can optimise sleep outcomes. Future research should prioritise well-controlled, large-scale longitudinal studies with repeated measures, accounting for factors such as age, sex, and socioeconomic status in both healthy and unhealthy populations across the lifespan. A key focus should be on uncovering the underlying mechanisms of the diet–sleep relationship while integrating dietary interventions with circadian biology. Such research would enhance our understanding of how nutrition could influence sleep and overall health, potentially leading to personalised, evidence-based interventions that could optimise well-being.
